# Curcumin-loaded nanoparticles: a novel therapeutic strategy in treatment of central nervous system disorders

**DOI:** 10.2147/IJN.S208332

**Published:** 2019-06-17

**Authors:** Hanie Yavarpour-Bali, Maryam Ghasemi-Kasman, Marzieh Pirzadeh

**Affiliations:** 1 Student Research Committee, Babol University of Medical Sciences, Babol, Iran; 2 Cellular and Molecular Biology Research Center, Health Research Institute, Babol University of Medical Sciences, Babol, Iran; 3 Neuroscience Research Center, Health Research Institute, Babol University of Medical Sciences, Babol, Iran

**Keywords:** curcumin, central nervous system diseases, nanoparticles, water solubility, bioavailability

## Abstract

Curcumin as a hydrophobic polyphenol is extracted from the rhizome of *Curcuma longa*. Curcumin is widely used as a dietary spice and a topical medication for the treatment of inflammatory disorders in Asia. This compound also possesses remarkable anti-inflammatory and neuroprotective effects with the ability to pass from the blood brain barrier. Based on several pharmacological activities of curcumin, it has been introduced as an ideal candidate for different neurological disorders. Despite the pleiotropic activities of curcumin, poor solubility, rapid clearance and low stability have limited its clinical application. In recent years, nano-based drug delivery system has effectively improved the aqueous solubility and bioavailability of curcumin. In this review article, the effects of curcumin nanoparticles and their possible mechanism/s of action has been elucidated in various central nervous system (CNS)-related diseases including Parkinson’s disease, Huntington disease, Alzheimer’s disease, Multiple sclerosis, epilepsy and Amyotrophic Lateral Sclerosis. Furthermore, recent evidences about administration of nano-curcumin in the clinical trial phase have been described in the present review article.

## Introduction

It has been estimated that up to 1.5 billion people worldwide are suffering from central nervous system (CNS) disorders. The most challenging of the CNS diseases are neurodegenerative diseases, attributed to age-related gradual decline in neurological function, often accompanied by neuronal death. It has been shown that several mechanisms such as protein aggregations, oxidative stress and neuroinflammation are involved in neuronal death and damage.^1^–^3^ Recently, the use of natural compounds such as curcumin have been proposed as an alternative and effective strategy in the treatment of neurodegenerative and neurological diseases.^4^ Curcumin is a hydrophobic polyphenol that is derived from the rhizomes of the *Curcuma longa*.^5^ It has been well documented that curcumin possesses a wide variety of important pharmacological activities including anticancer,^6^–^9^ antimicrobial,^10^ anti-inflammatory,^11^ anti-amyloid,^12^,^13^ antioxidant,^14^ and neuroprotective effects.^15^ Traditionally, turmeric has been used for several aliments and especially it is widely consumed for dietary and medicinal purposes in Southeast Asia, the China, and India.^16^,^17^ It can also cross the blood-brain barrier (BBB) and due to its pleotropic therapeutic effects, curcumin has been regarded as a potential therapeutic factor for a large number of nervous system diseases.^5^ In addition, curcumin has vast application in the treatment of many other diseases such as cancer, diabetes, cystic fibrosis, malaria, and hypertension.^18^,^19^ Due to the pleotropic actions of curcumin on the nervous system, it could be regarded as a potent neuroprotective compound in the treatment of CNS-associated diseases.^20^ Beneficial effects of curcumin in neuroinflammatory diseases including Alzheimer’s disease (AD),^21^ Multiple sclerosis (MS),^22^ and Parkinson’s disease (PD)^23^ have been well documented in several experimental and clinical studies.

Despite the remarkable pharmacological activities of curcumin, low aqueous solubility‚ poor stability in the body fluids‚ high rate of metabolism‚ rapid clearance, reduced absorption in gastrointestinal (GI) tract and limited bioavailability has hampered the clinical application of curcumin.^5^,^17^ Although, a large number of clinical trials using curcumin and its classification have confirmed the safety of curcumin, the afore-mentioned obstacles are the main reasons that it has not yet been approved as a drug for clinical application.^24^ In recent years, numerous approaches including usage of adjuvant molecules like piperine‚ quercetin or silibinin‚ application of structural analogues of curcumin‚ chemical complex of curcumin with phospholipids, polysaccharides or proteins, and bio conjugates of curcumin with turmeric oil or alanine have been employed to increase the solubility and bioavailability of curcumin.^25^,^26^ Although the above-mentioned strategies increase the solubility and bioavailability of curcumin, most of these formulations cannot protect curcumin completely and it could be rapidly metabolized from the body. In addition, these strategies do not provide an effective way for targeting of curcumin into specific sites of action.

**Figure 1:**
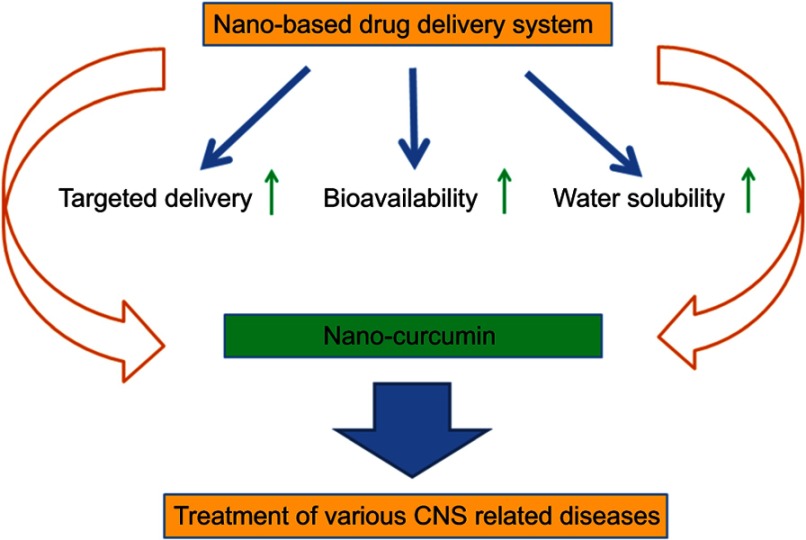
Nano-based drug delivery system for targeted delivery and enhancement of water solubility and bioavailability of curcumin. **Abbreviation:** CNS, Central nervous system.

During the past decades, researchers have investigated the beneficial effects of various nanomaterials in drug delivery, targeted therapy, and imaging processes.^27^ Interestingly, nanoparticle-based delivery systems have been emerged as a novel approach to improve the water solubility and enhance the bioavailability of therapeutic agents such as curcumin (Figure 1). It has been shown that encapsulation of curcumin in nanoparticles considerably improves the chemical stability of curcumin and prevents its enzymatic and pH degradation. Additionally, the formulation of curcumin in nanoparticles increases its circulation inside the body.^5^,^17^,^28^ Accordingly over the last decades, numerous nanoformulation-based strategies have been undertaken to improve the properties of curcumin in in vitro, in vivo, and pre-clinical settings. Nanoformulation-based strategies involve the use of adjuvants, stabilizers, conjugates/polymer conjugates, lipid/liposomes, hydro/micro/nano gels, micelles, and nanoparticles (NPs).^29^–^31^

In this review article, the current literature about the effect of nano-curcumin on major neurological diseases such as AD, PD, Huntington disease (HD), MS, epilepsy and Amyotrophic Lateral Sclerosis (ALS) have been addressed. In addition, recent studies on the potential efficacy of nano-curcumin in clinical application have been discussed.

### Application of nano-curcumin in AD

The main pathological features of AD include aggregation of extracellular amyloid plaques and intracellular neurofibrillary tangles (NFTs) and hyper-phosphorylated tau protein. Although, the current hypotheses emphasize different stories including β-amyloid deposit, tauopathy, oxidative stress, calcium overload, cholinergic, and glutamatergic neurotransmission alterations. β-amyloid cascade and tauopathy are still the most widely accepted central factors triggering and/or accelerating the AD pathogenesis.^32^ Several experimental reports demonstrated the potential effects of curcumin in AD treatment by regulating multiple pathways such as reduction of inflammation, activation of neurogenesis and Aβ inhibition.^33^,^34^ Recently, curcumin nanoparticles have emerged as a novel strategy for treatment of AD.^35^ Tiwari et al^35^ have investigated the effect of curcumin-loaded nanoparticles for its ability to induce adult neurogenesis. They explored the neuroprotective efficacy of curcumin-encapsulated biodegradable poly (lacticco-glycolic acid) (PLGA) nanoparticles (NPs) on neural stem cells (NSCs) proliferation and differentiation both in vitro and in vivo. Several neurogenic genes/transcription factors such as neurogenin, neuregulin, neurogenic differentiation 1 (neuroD1), neuroligin, and signal transducer and activator of transcription 3 (Stat3) were increased after Cur-PLGA-NPs treatment. For the first time, it has been also found that reelin, nestin, and Pax6, a panel of genes concerned with neuronal proliferation and self-renewal, were up-regulated by Cur-PLGA-NPs. Wnt/β-catenin signaling regulates adult hippocampal neurogenesis and is involved in self-renewal of NSC/progenitor cells. Based on their study, Cur-PLGA-NPs increased gene expression and protein levels of Wnt due to decrement of Wif-1, Dkk-1, and GSK3β, (negative regulators of Wnt pathway). Cur-PLGA-NPs also reversed learning and memory deficit in an amyloid beta-induced rat model of AD-like phenotypes. Overall, results of this study suggested that curcumin nanoparticles may provide a novel therapeutic point for the treatment of neurodegenerative diseases.^37^ Additionally, it has been shown that selenium nanoparticles encapsulated PLGA nano spheres with curcumin are regarded as a novel nanoformulation for treatment of AD. Investigating the effect of this drug delivery system in transgenic mice demonstrated the inhibitory effect of nano spheres on Aβ aggregation and toxicity as well as decreasing inflammation in AD pathogenesis.^38^ In another study, Meng et al^39^ designed and engineered a novel low density lipoprotein (LDL)-mimic nanostructured lipid carrier (NLC) modified with lactoferrin (Lf)-loaded curcumin and evaluated the potency of novel synthesized nano-curcumin for brain-targeted delivery and its regulatory effect on progression of AD. The results revealed that curcumin nanoformulation was able to cross the BBB and release curcumin in an effective manner. To examine the effect of Lf_H_-mNLC-cur on neuronal loss, Aβ_1-42_ was bilaterally injected into the dorsal hippocampus. Histopathological measurements revealed the ameliorating effect of Lf_H_-mNLC-cur on neuronal damage. Blood samples also were taken to investigate the plasma levels of malondialdehyde (MDA), an important indicator of lipid peroxidation. The result showed a decrement in the level of MDA in cur-formulation treated groups. This finding demonstrated that Lf_H_-mNLC group could successfully cross the BBB and effectively decrease the damage associated with oxidative stress. According to the mentioned results, they postulated that Lf_H_-mNLC-cur can be considered as an effective drug for brain-targeted delivery. Another novel engineered nanoformulation had been designed by Barbara et al.^42^ Curcumin-encapsulated PLGA nanoparticles bounded to cur-NPs-g7 and were tested for its toxicity, targeted delivery, and biological activity using an in vitro model of AD. Cur-NPs-g7, that is able to cross the BBB, was led to a reduction in oxidative stress level, inflammation and Aβ aggregation. CellRox^®^ staining revealed that cur-NPs-g7 decreased the levels of oxidative stress in treated cells by its free radical scavengering effects. In addition, cur-NPs-g7 prevented the reduction of IκB (NF-κB inhibitor protein family) which was observed in the presence of Aβ aggregation.^40^ Another anti-inflammatory mechanism of this formulation could be manifested from a report which proposed that curcumin can bind to copper and zinc ions.^41^ Moreover, cur-NPs-g7 formulation was detected as Aβ aggregation inhibitor and Aβ disaggregation activator. Taken together, the results of this study indicated that cur-NPs-g7 is an effective medication for treatment of AD.^42^ Currently, a brain targeting cyclic CRTIGPSVC peptide (CRT)-conjugated PLGA NP loaded with Aβ generation inhibitor S1 and curcumin (as a therapeutic agent to tackle the disorder) was fabricated. CRT peptide up-regulated the permeation of PLGA NPs across an in vitro BBB model, which was built up by microvascular bEnd3 cells. The polymeric system significantly decreased Aβ deposit, microglial activation and astrogliosis which are involved in pathogenic process of AD.^43^ It has been shown that after treatment byNP-S1, NP-Cur, NP-S1+Cur or CRT-NP-S1+Cur; the number of activated glial cells was significantly reduced. Furthermore, PLGA NPs increased the number of synapses, prevented inflammation by decreasing of pro-inflammatory cytokines (IL-6 and TNF-α) and restored antioxidant activity via decreasing the ROS level and increasing the level of SOD in the mouse brain. This new formulation of nano-curcumin not only affected brain’s neuropathy but also attenuated behavioral deficits by increasing the spatial memory and recognition.^44^ Cheng et al's^35^ report demonstrated that oral administration of curcumin-loaded polyethylene glycol-polylactic acid co-block polymer (PEGPLA NPs) in Tg2576 mice for 3 months remarkably enhanced memory in the contextual fear conditioning test and working memory in the radial arm maze test. Furthermore, antioxidant and anti-inflammatory effects of curcumin-loaded PLAG nanoparticles on the expression of key genes involved in neuroprotection and antioxidant pathways were studied in an in vitro model on human neuroblastoma cell line (SK-N-SH cells). Curcumin-loaded PLAG nanoparticles increased the expression of GLRX, TRX (the key components of the thiol redox buffering system) and decreased APOJ (which is overexpressed in neurodegeneration) and had no effect on APOE and REST expressions. The effects of curcumin-loaded nanoparticles on three signaling pathways (Nrf2, NF-*k*B and Akt/pTau) were in accordance with its anti-inflammatory and antioxidant properties. The above-mentioned pathways are involved in the regulation of oxidative stress, inflammation, neuronal survival, and phosphorylation of tau protein. Hence, cur-NPs prevented Akt activity and Tau phosphorylation, as well as suppressed activation of Nrf2 and NF-κB signaling pathways; the efficacy of curcumin-loaded PLAG nanoparticles was more potently compared to the free curcumin. Additionally, higher antioxidant capacity against peroxyl radical than free curcumin and blank was reported for curcumin-loaded PLAG nanoparticles. Overall, this study offered PLGA nanoparticles as a promising tool for the delivery of drugs in treatment of AD.^45^ Fan et al^48^ also prepared curcumin-loaded PLGA-PEG nanoparticles conjugated with B6 peptide (PLGA-PEG-B6/Cur). The in vitro assays including dynamic light scattering (DLS), flow cytometry (FCM), red blood cell (RBC) lysis, and thromboelastographic (TEG) analysis showed good bio-safety and high bioavailability of PLGA-PEG-B6/Cur. Furthermore, the results indicated that administration of PLGA-PEG-B6/Cur into HT22 cells was in agreement with mentioned in vitro assays. In accordance with previous studies about different properties of cur in AD pathogenesis including high Aß affinity, anti-β amyloidosis, and anti-tauopathy,^46^,^47^ the efficacy of PLGA-PEG-B6/Cur was investigated in APP/PS1 Al transgenic mice. Treatment with PLGA-PEG-B6/Cur was led to a decrement in the level of Aß and tau phosphorylation and inhibited the generation of BACE1, APP, and PS1as markers of Alzheimer’s disease. Additionally, enhancing spatial learning and memory capability of APP/PS1 mice was another effect of PLGA-PEG-B6/Cur. In conclusion, the results of the current study illustrated that PLGA-PEG-B6/Cur might be considered as a promising therapeutic strategy for the treatment of AD in the future.^48^

### Application of nano-curcumin in PD

Oxidative stress plays a key role in the pathology of PD including several degeneration reactions such as nitric oxide and mitochondrial toxicity.^49^,^50^ It has been demonstrated that nano-curcumin can significantly reduce the oxidative stress and apoptosis in the brain of PD files.^51^ Similarly, encapsulation of curcumin in alginate nanoparticles enhanced the neuroprotection through reducing of oxidative stress and brain cell death in a transgenic Drosophila PD model.^52^ The results indicated that the bioavailability of nano-curcumin was higher in mouse brain and had a protective effect against the oxidative stress. Bollimpelli et al^53^ elaborated lactoferrin nanoparticles by sol-poil chemistry which was loaded with curcumin (LF-NP-cur). Neuroprotective effect of LF-NP-cur was evaluated against rotenone-induced toxicity in SK-N-SH cells. It was reported that the expression of lactoferrin receptors, which have a role in iron uptake by cells increases in PD patients. Based on this reason, lactoferrin can play an important role in specific targeting of curcumin. It has been shown that LF-NP-cur reduced the level of ROS, which was induced by rotenone in SK-N-SH cells. Moreover, expression of α-synuclein was greatly decreased in LF-NP-cur cells. In another study, the exposure of PD files to alginate-curcumin nanocomposite (ACNC) revealed a marked delay in the climbing ability. Curcumin has been reported to reduce the inflammation and oxidative stress in many degenerative disorders. It can also exhibit protective effect against α-synuclein-induced cytotoxicity in SH-SY5Y neuroblastoma cells by reducing the cytotoxicity of accumulated α-synuclein, decreasing intracellular reactive oxygen species (ROS), preventing the activation of caspase 3 and reducing apoptosis. Additionally, results of this study clarified that ACNC can markedly postpone the loss of climbing ability of the animals in a PD-induced model and decreased oxidative damage as well as apoptosis in the brain of animals. It has been shown that antioxidant effect of ACNC was mediated through the reduction of lipid peroxidation. In addition, it has been suggested that reduction of lipid peroxidation and apoptosis in the brain of animals could be due to the suppressive effect of ACNC on α-synuclein accumulation or result from the property of curcumin to scavengering free radicals.^54^ Another study documented that curcumin-loaded polysorbate 80-coated cerasome (CPC) nanoparticles hold the potential to moderate the release time of curcumin and produced a prolonged circulation time in the blood. Furthermore, CPC was combined with ultrasound-targeted microbubble destruction (UTMD) to locally open the BBB around the targeted brain nuclei. Administration of ps 80-NP-cur with UTMD exhibited remarkable reduction of PD symptoms in PD mouse model induced by 1-methyl-4-phenyl-1, 2, 3, 6, tetrahydropyridine (MPTP).^55^ In another study undertaken by Kundu et al,^56^ piperine and curcumin were loaded into glyceryl monooleate (GMO) nanoparticles. GMO-NP-Pip/Cur remarkably inhibited aggregation of α-synuclein into oligomers and fibrils. The anti-oxidant and anti-apoptotic activities of GMO-NP-Pip/cur were investigated with no evidence of cytotoxicity. GMO-NP-Pip/Cur attenuated motor dysfunction via decreasing the oxidative stress, apoptosis and enhancing the autophagy. Higher density of tyrosine hydroxylase (TH) positive neurons was observed in GMO-NP-Pip/Cur receiving animals. Employing TH gold standard marker with dual drug-loaded NPs revealed more intense effect of GMO-NP-Pip/Cur in protecting of dopaminergic neurons against rotenone-induced degeneration model.^56^ Currently, another study showed that bovine serum albumin (BSA)-based nano-curcumin can be regarded as a potential therapeutic strategy against 6-OHDA-induced cell death in SH-SY5Y cells as a cellular model of PD. It has been suggested that cell death might result from oxidative stress and ROS generation and nano-curcumin prevents cell death via it’s antioxidant properties.^57^ Recently, a report by Rakotoarisoa et al^58^ showed the neuroprotective effect of another nanoformulation of curcumin including curcumin and fish oil-loaded spongosome and cubosome nanoparticles in SH-SY5Y cells. The results revealed the neuroprotective potential of this compound against ROS accumulation and H_2_O_2_-induced cell death. Akt signaling has been introduced as one of the main mechanisms involved in cell survival^59^,^60^ and it is commonly dysregulated in PD patients.^61^ However, treatment with this cur-nanoformulation in protective doses up-regulates the p-Akt/t-Akt signaling pathway. Based on the above-mentioned results, it has been suggested that BSA-based nano-curcumin can be considered as an alternative therapeutic agent in neurodegenerative disease.^60^

### Application of nano-curcumin in HD

Sandhir et al^62^ have developed a formulation of curcumin (Cur-SLN) with enhanced oral bioavailability and investigated its potential therapeutic impact in 3-nitropropionic acid-induced-(3-NP) HD model in rats. The results indicated that Cur-SLN administration affected mitochondrial complexes activities by increasing the activity of NADH dehydrogenase, SDH activities as well as cytochrome oxidase. They evaluated the effect of Cur-SLN on 3-NP-induced alterations in mitochondrial integrity and cytochrome levels. Their data revealed significantly increased levels of various cytochromes including cytochrome a, b, c1 and c. Regarding mitochondrial integrity, considerable reduction in mitochondrial swelling was also observed. Moreover, the levels of mitochondrial oxidative stress markers including MDA, protein carbonyl and ROS were diminished and the levels of GSH and SOD as antioxidant enzymes were increased in Cur-SLN treated animals. Furthermore, administration of Cur-SLN increased the expression of Nrf2. Investigating the effect of Cur-SLN on neurobehavioral deficits was another approach, which was measured in this study. The experimental results indicated that Cur-SLN significantly increased the locomotor activity. In conclusion, they proposed that Cur-SLN is considered as a promising strategy to attenuate the mitochondrial impairments in 3-NP-induced HD model and also might be useful in other neurodegenerative disorders involving mitochondrial dysfunctions, oxidative stress and inflammation.^62^

### Applicationn of nano-curcumin in MS

MS is a chronic inflammatory autoimmune disease of the CNS which is characterized by neurodegenerative processes.^63^ Two major aspects of MS are acute inflammation that is associated with demyelination and axonal loss. At the present time, most of the strategies in treatment of MS have focused on preventing inflammation in the CNS.^64^ It has been reported that nano-curcumin has great potential for treatment of MS.^65^ In an experimental autoimmune encephalomyelitis (EAE) model of MS, polymerized nano-curcumin (PNC) decreased scores of disease in therapeutic and contraceptive administration. In addition, expression of pro-inflammatory factors were reduced in PNC-treated animals. Conversely, the expression of anti-inflammatory genes such as IL-4 ‚IL-10 and TGF-ß was increased in animals under treatment of PNC. Similary, application of PNC increases the level of FOXP3 as a regulatory transcription factor of T regulatory cell which modulates the production of T helper cytokine. Furthermore, it has been illustrated that PNC significantly increases the expression of HO-1 gene through inducing of Nrf2 signaling pathway while diminishes the mRNA level of iNOS. In addition, the results of this study demonstrated the myelin basic protein (MBP) expression level in treated group was even higher than intact group. Furthermore, their data showed the enhanced expression level of nestin‚ a marker of neural stem cell as well as Olig2 and platelet-derived growth factor-alpha receptor (PDGFR-α), as oligodendrocyte progenitors markers. Therefore, it seems that PNC through up-regulation of oligodendrocytes production increases the level of MBP. Another possible mechanism of PNC in improvement of remyelination was related to increasing brain-derived neurotrophic factor (BDNF) and nerve growth factor (NGF) levels following PNC treatment.^66^ In a current study by Naeimi et al,^67^ it has been shown that curcumin-loaded nanoparticles ameliorate glial activation and augment myelin repair in lyolecthin-induced focal demyelination model of rat corpus callosum. Based on the results of this study, it has been demonstrated that curcumin-loaded nanoparticles caused significant decrement in immune cells infiltration and reduced the extent of demyelination areas and these effects was more remarkable compared to the free curcumin. In addition, the levels of glial activation including GFAP (as astrocyte marker) and Iba1(as microglia/macrophage marker) significantly attenuated in curcumin-loaded nanoparticle-receiving animals.

### Application of nano-curcumin in epilepsy

Epilepsy is a chronic neurological disorder characterized by recurrent unprovoked seizures which affects 1% of the world’s population.^68^, Several lines of evidence showed the pro mising anticonvulsant effect of curcumin.^69^,^70^ It has been shown that curcumin nanoparticles (cur-NPs) possess antioxidant‚ anti-inflammatory‚ anti-apoptotic and anti-convulstant activities in pilocarpine-induced status epilepticus (SE) model in rats.^71^ The findings of this study demonstrated that cur-NPs prevented the increased level of lipid peroxidaton and hippocampal level of NO as well as improved the cortical level of GSH which were decreased after induction of SE. Further more, cur-NPs decreased the level of TNF-α in the hippocampus. In agreement with this report, another study has also illustrated that curcumin nanoformulation can reduce the level of inflammatory factors such as TNF-α and IL-1ß in middle cerebral artery occulusion–induced cerebral ischemia in rats.^72^ Cur-NP played its role as an anti-apoptotic agent by downregulating of caspase 3. Aminirad et al^73^ reported that acute administration of nano-curcumin exhibited dose-dependent anticonvulsant activities against the seizures. The results of this study suggested that one of the probable anticonvulsant mechanisms of nano-curcumin to raise the latency to PTZ-induced clonic seizures is reduction of inducable NO synthase inhibitor (iNOS) expression which is led to NO downregulation.^73^ In parallel with the mentioned reports, it has been documented that liposomal formulation of curcumin (25 and 50 mg/kg) dose dependently elevated the seizure threshold and latancy to PTZ-induced myoclonic jerk and generalized tonic-clonic seizures (GTCS). Additionally, liposomal formulation of curcumin increased the latency period and decreased the duration of clonic seizures during status epilepticus model of epilepsy.^74^ In another study, Rostami et al^75^ showed that there is a correlation between neuronal cell death and Klotho/erythropoietin (EPO) expression in an experimental model of chronic epilepsy. Their data clarified significant attenuation of neuronal cell death following treatment with curcumin-loaded nanoparticles as well as remarkable upregulation of Klotho and EPO. Furthermore in animals that had undergone curcumin-loaded NPs treatment, the mRNA level of TNF–α was considerably decreased. Based on these results, it has been postulated the neuroprotective effects of curcumin-loaded nanoparticles might be a result of downregulation of TNF–α and subsequent upregulation of klotho and EPO.^75^ Hashemian et al^76^ has reported that encapsulation of curcumin onto chitosan-alginate-sodium tripolyphosphate nanoparticles (CS-ALG-STPP NPs) showed anti-epileptogenic effect by decreasing seizures stage and duration of GTCS. It has been also shown that curcumin-loaded NPs could attenuate memory deficits, glial activation and cell loss following PTZ adminstration. According to the results of this study, it has been suggested that curcumin-loaded CS-ALG-STTP NPs might be considered as an effective therapeutic approach in treatment of epileptic patients.^76^

### Application of nano-curcumin in ALS

Mesenchymal stromal cells (MSCs) have opened a new horizon in enhancement of CNS repair. MSCs are able to migrate to the damaged tissues and exhibit the potential to cross the BBB.^77^,^78^ Actually, the importance of MSCs in the treatment of CNS diseases such as ALS was also demonstrated through enhancing neural protection and replacing dead motor neurons of the spinal cord in a study accomplished by Tripodo et al.^79^ In this study, insulin-d-alpha-tocopherol succinate micelles (INVITE)-loaded-curcumin micelles were used in order to improve the therapeutic effect of MSCs. This approach can guarantee an accurate drug targeting mediated by the innate ability of MSCs to reach injured tissues while protecting the incorporated drug from premature release or preventing toxic effects to MSCs. The afore-mentioned innovative drug delivery system has been proposed as an ideal strategy for the treatment of neurodegenerative diseases such as ALS.^79^

### Application of nano-curcumin in clinical trials

Currently, several clinical trials are in progress that are expected to provide an even deeper understanding of the therapeutic effects of curcumin.^65^ Dolati et al^80^ evaluated the anti-inflammatory effect of nano-curcumin in relapsing-remitting MS (RRMS) patients. In their study, 50 MS patients were randomly assigned into two groups. One group received a nano-curcumin capsule daily for 6 months and at the same time, the control group received placebo. Blood samples were collected and miRNAs expression levels, miRNA-dependent targets, expression of transcription factors and pro-inflammatory cytokines were evaluated in blood samples. The results showed the levels of some miRNAs including miR-132, miR-16 and miR-145 were reduced and levels of their targets including sitrulin1, Sox2, Foxp3 and PDC1, respectively elevated. Moreover, the expression levels of STAT1, NF-κB, AP-1, IL-1β, IL-6, IFN–γ, CCL2, CCL5 and TNF-α were significantly down-regulated. Additionally, the EDSS measurement showed considerable decrement in the nano-curcumin-treated group.^80^ In another randomized, double blind, placebo-controlled trial, 50 MS patients participated and were divided into two groups. Twenty-five subjects received curcumin containing nano-micelles capsules, and the control group received placebo. Blood samples were collected at two different times. One of them was collected before administration of either nano-curcumin capsules or placebo and the other was taken after 6 months. The current study validated the possible therapeutic effects of nano-curcumin on the expression levels of some microRNAs in peripheral blood mononuclear cells (PBMCs) of MS patients. As reported by previous studies, an abnormal pattern of miRNAs level was observed in MS patients.^81^ Furthermore, it has been found that miRNAs have contributed to the pathogenesis of MS via multiple factors and pathways^82^–^84^ which affects immune cells spatially (B and T cells). Gene expression analysis revealed the expression level of microRNAs including miR-16, miR-17-92, miR-27, miR-29b, miR-126, miR128, miR-132, miR-155, miR-326, miR-550, miR-15a, miR-19b, miR-106b, miR-320a, miR-363, miR-31, miR-181c, miR-150, miR-340, and miR-599 moderated after nano-curcumin treatment. Accordingly, the expression of miR-16, miR-17-92, miR-27, miR-29b, miR-126, miR-128, miR-132, miR-155, miR-326, miR- 550, and miR-340 was significantly diminished in nano-curcumin-receiving RRMS patients while the expression of miR-15a, miR-16, miR-19b, miR-106b, miR-320a, miR-363, miR-31, miR-181c, miR-150, miR-340, and miR-599 was considerably increased in these patients. In conclusion, the authors suggested that nano-curcumin might be regarded as a beneficial therapeutic agent for treatment of MS patients and also as an immunomodulatory compound.^85^ Th17 cells are at the center of attention in chronic inflammatory diseases researches^86^–^88^ and it has been reported that curcumin suppresses Th17 differentiation and its related pathways.^89^,^90^ In accordance to these studies, Dolati et al evaluated the frequency of Th17 cell-related cytokines expression level in MS patients. This clinical trial was accomplished with 35 healthy controls, 25 patient subjects who received nano-curcumin, and 25 patients who received placebo. Experimental procedures were performed at two different times for the nano-curcumin group, one at baseline and the other 6 months after nano-curcumin treatment. The results exhibited that the ratio of Th17 cells were greatly reduced after treatment with nano-curcumin. Moreover, mRNA expression level of IL-17 significantly decreased while there was no change in IL-23 mRNA expression after nano-curcumin administration. They also investigated the secretion levels of IL-17 and IL-23 in MS patients and their data indicated that nano-curcumin significantly reduced the levels of IL-17and IL-23. Likewise, nano-curcumin treatment considerably decreased the total EDSS. They illustrated that nano-curcumin may be a promising neuroprotective compound with the ability to regulate inflammation in MS.^91^ Dysfunction of T-regulatory (Treg) cells are considered to have a basic role in autoreactive immune response in MS.^92^ The efficacy of nano-curcumin in Treg frequency and function were assessed in a randomized, double blind, placebo-controlled trial study. Fifty patients with RRMS were enrolled in this study and randomly assigned to receive either a nano-curcumin capsule or a placebo in a 1:1 ratio. Blood samples were collected at baseline and 6 months after receiving nano-cur capsules or placebo. The results indicated that nano-curcumin administration could increase the frequency of Treg lymphocytes. Furthermore, cytokine expression analysis showed a significant elevation in the level of Treg-associated cytokines including TGF-β and IL-10. Similarly, the expression level of FOXP3 was up-regulated in PHA-stimulated Treg cells. In line with the previous mentioned reports, the total EDSS was significantly decreased following nano-curcumin treatment. According to the above-mentioned results, nano-curcumin plays an important role as an immunomodulatory agent and prevents auto reactivity through its effect on frequency and function of Treg cells.^93^ A double-blind, randomized, placebo-controlled trial was also performed to investigate the efficacy of nano-curcumin (80 mg/kg) in patients with ALS. The survival probability of patients with ALS was significantly improved over the 12-month period of this study. No significant difference was observed in ALSFRS-R score, muscle strength, and CMAP amplitude decrements between the nano-curcumin and placebo groups.^94^

## Limitations and future prospects

The above-mentioned studies indicated that nanoformulation of curcumin markedly improves its efficacy and bioavailability in in vitro and in vivo conditions. Tables 1 and 2 summarize the current literature about application of curcumin nanoparticles both in vitro and in vivo. However, more studies are required to investigate the toxicity and efficacy of curcumin-loaded NPs in large group of patients. Furthermore, combination therapy with curcumin-loaded NPs is appropriate for decreasing the dose of the main therapeutic agent, which can result in improved therapeutic efficacy while reducing systemic toxicity. Additionally, it should be noted that although functionalized nanoparticles represent successful drug targeting, their nano-size structure and the large surface area may lead to particle aggregation and limited drug loading.^95^,^96^ State of aggregation and mechanical properties affects nanoparticles toxicity, which depends on preparation and purification methods. Hence, further studies are required to prepare curcumin-loaded NPs with lower toxicity. Toxicity concerns of nanomedicine-based delivery systems includes that it can lead to neuroinflammation, excitotoxicity, DNA damage, and allergic responses.^97^ Therefore, biocompatibility and biodegradability of nanodrugs should be precisely studied.

**Table 1:** In vitro application of nano-curcumin

Nano-Cur	Cellular model	Disease	Key Finding	Ref.
Curcumin-loaded PLAG NPs	Neuroblastoma cell line (SK-N-SH cells)	AD	Inhibition of Nrf2, NF-*k*B and Akt phosphorylation Tau/↓ Expression of APOJ/↑ Expression of GLRX and TRX	45
g7-NPs-Cur	Primary hippocampal cell cultures/Aß _(1–42)_	AD	↓ Oxidative stress/↓ Inflammation/↓NF-kB/↑IkB//↑ Aß disaggregation	42
PLGA-PEG-B6/Cur	HT22 cells	AD	Narrowing the diameter of Cur/↑Cellular uptake	48
Alginate-curcumin nanocomposite (ACNC)	α-synuclein-induced cytotoxicity in SH-SY5Y neuroblastoma cells	PD	Apoptosis↓/Postpone the loss of climbing ability/Preventing caspase 3 activation/↓ Oxidative damage/↓ Cytotoxicity of accumulated α-synuclein	54
LF-NP-Cur	Rotenone-induced toxicity in SK-N-SH cells	PD	↓ ROS levels/Suppression of α-synuclein expression/↑ Expression of TH enzyme	53
GMO-NP-Piperine/Curcumin	Dopaminergic neurons against rotenone-induced degeneration model	PD	↓ Aggregation of α-synuclein/↓ Motor dysfunction/↓ Oxidative stress/↓ Apoptosis/Enhancing the autophagy/↑ Density of TH positive neurons	56
BSA-based nano-curcumin	6-hydroxydopamine (6-OHDA)-induced death and Akt signaling disruption in SH-SY5Y cells	PD	Corrected p-Akt/t-Akt signaling/prevent cell death	60
Curcumin and fish oil-loaded spongosome and cubosome NPs	H_2_O_2_-induced oxidative stress in differentiated human SH-SY5Y cells	PD	↓ H_2_O_2_-induced cell death/ ↓ ROS accumulation	58
Insulin-d-alpha-tocopherol succinate micelles (INVITE)–loaded curcumin micelles	Mesenchymal stromal cells (MSCs)	ALS	Maximum loading in MSCs/no cytotoxicity	79

**Abbreviations:** PLGA, Poly (lactic-co-glycolic acid); NPs, Nanoparticles; AD, Alzheimer’s disease; Cur, Curcumin; PEG, Polyethylene; PD, Parkinson’s disease; LF, Lactoferrin; ROS, Reactive oxygen species; TH, Tyrosine hydroxylase; GMO-NP, Glyceryl monooleate-nanoparticle; BSA, Bovine serum albumin; ALS, Amyotrophic lateral sclerosis.

**Table 2:** In vivo application of nano-curcumin in CNS related disorders

Nano-Cur	Animal model	Disease	Key findings	Ref.
PLGA (2 mg/kg)	APP/PS1dE9 mice	AD	↓ Aß_42_/↓ Aß _40_/↑ SOD ↓ TNF-α/↓ IL6/↓ROS/↑ Synapse number/Inhibit APP cleavage/Suppress microgliosis and astrogliosis	83
Cur-PLGA-NPs (5,10, and 20 mg/kg)	Aβ-induced rat model of AD	AD	↑ Pax6 and reelin expression/↑ NSCs proliferation, Self-renewal, Neuronal differentiation/↓ Expression of Axin12, APC, GSK-3ß/↑ Cyclin-D1 and TCF/LEF Promoter activity expression of Wnt3, LRPP-5, LEF/↓ Expression of Wif-1,Dkk-1/↑ p-GSK-3β cells/↓ p-β-catenin cells/↑GSK-3β phosphorylation/↓ phosphorylation of β-catenin	37
Selenium/Curcumin-PLGA nanosphere	Transgenic mice 5XFAD	AD	↓ Aß aggregation and toxicity	38
Low density lipoprotein mimic nanostructured lipid carrier modified with lactoferrin-loaded curcumin (6 mg/kg)	Administrating Aβ_1-42_ and D-gal in rats	AD	↓ MDA level/↓ Damage associated with oxidative stress/↓ Lipid peroxidation	39
CRT-conjugated PLGA 2 mg/kg (i.p.)	AD transgenic mice	AD	↓ Activated glial cell/↑ Number of synapses/↓ IL-6, TNF-α/Restore antioxidant activity (↓ ROS)/↑ SOD/↑ Spatial memory/Enhanced behavioral deficit/Suppress astrogliosis and microgliosis	44
Curcumin-loaded PEG-PLA NPs (23 mg/kg)	Tg2576 mice	AD	Enhanced cue memory	35
PLGA-PEG-B6/Cur	APP/PS1 mice	AD	Enhance the spatial learning and memory capability/Inhibit the generation of BACE1, APP, and PS1/↓ tau phosphorylation and Aß aggregation	48
Alginate-curcumin nanocompposite	Transgenic Drosophila model	PD	↑ Bioavailability/↓ Oxidative stress/↓ Apoptosis	54
Curcumin-loaded polysorbate 80-coated cerasome NPs with UTMD (15 mg curcumin/kg)	PD mouse model induced by 1-methyl-4-phenyl-1, 2, 3, 6, tetrahydropyridine (MPTP)	PD	↓ PD symptoms/↑ Circulation time in blood	55
Curcumin encapsulated solid lipid NPs (40 mg/kg)	In 3-nitropropionic acid-induced-(3-NP) Huntington’s disease model in rats	HD	Enhance mitochondrial activity (↑ activity of NADH dehydrogenase/↑ SDH activities/↑ cytochrome oxidase)/↓ Mitochondrial swelling/↓ MDA, protein carbonyl and ROS/↑ GSH and SOD enzymes/↑ Expression of Nrf2/↑ Locomotor activity	62
Polymerized nano-curcumin (12.5 mg/kg)	Experimental autoimmune encephalomyelitis (EAE) model of MS	MS	↑ IL-4 ‚IL-10‚ TGF-ß/↑ FOXP3/↑ Expression of HO-1 gene/Induction of Nrf2 signaling pathway/↓ iNOS mRNA level/↑ MBP expression level/↑ Expression levels of nestin, Olig2 and PDGFR-α/↑ BDNF and NGF levels	66
Curcumin-loaded NPs (12.5 mg/kg, i.p.)	Lysolecthin-induced focal demyelination model of rat corpus callosum	MS	↓ Glial activation/↑ Myelin repair/↓ Immune cells infiltration /↓ Extent of demyelination areas	67
Curcumin NPs (20, 40, 80 mg/kg,) (i.p.)	Pilocarpine-induced status epilepticus (SE) model in rats	Epilepsy	↓ Lipid peroxidation/↑ GSH and NO/↓TNF-α/↓ Caspase 3/↓ iNOS expression/↑ Latency to PTZ-induced clonic seizures	73
Liposomal formulation of curcumin(25 and 50 mg/kg)	Status epilepticus model in mice	Epilepsy	↑ Latency to PTZ-induced myoclonic jerk and generalized seizures/↓ Duration of clonic seizures	74
Curcumin-loaded NPs (12.5 mg/kg, i.p.)	Pentylenetetrazol (PTZ)-induced kindling model	Epilepsy	↓ Neuronal cell death/↑ Levels of EPO and klotho/↓ mRNA level of TNF-α	75
Chitosan-alginate-sodium tripolyphosphate nanoparticles (12.5 or 25 mg/kg, i.p.)	PTZ-induced kindling model	Epilepsy	↑ Spatial learning and memory/↓ Cell death and glial activation	76

**Abbreviations:** PLGA, Poly (lactic-co-glycolic acid); NPs, Nanoparticles; APP, Amyloid precursor protein; AD, Alzheimer’s disease; SOD, Superoxide dismutase; ROS, Reactive oxygen species; NSCs, Neural stem cells; PEG-PLA, Polyethylene glycol- polylactic acid; PD, Parkinson’s disease; HD, Huntington’s disease; MS, Multiple sclerosis; SDH, Succinate dehydrogenase; MDA, Malondialdehyde; GSH, Reduced glutathione; iNOS, Inducible nitric oxide synthase; MBP, Myelin basic protein; PDGFR, Platelet-derived growth factor receptor; BDNF, Brain-derived neurotrophic factor; NGF, Nerve growth factor.

## Conclusion

Curcumin has an outstanding safety profile and exerts a number of pleiotropic activities including anti-inflammatory, antioxidant, and anti-protein aggregate effects. Additionally, it is regarded a safe and inexpensive compound, readily available and useful as it can effectively penetrate into the BBB and neuronal membranes. However, low aqueous solubility, rapid clearance and poor stability in the body fluids limit its clinical application. Currently nano-based drug delivery systems are opening a new horizon to tackle the afore-mentioned problems. In the present study, we have documented a number of in vitro, in vivo, and clinical trial reports, which have provided evidence for the bioactive role of curcumin NPs in the prevention and treatment of various CNS-related diseases. Different formulations of nano-curcumin efficiently tackled various signaling pathways that are linked to different neurological disorders. We can also predict that drug delivery using nanotechnology can revolutionize the era of traditional drug delivery systems and modified drugs will be extremely efficient compared to the current standard.
